# Correction: Mesnard et al. Eliminating Synaptic Ribbons from Rods and Cones Halves the Releasable Vesicle Pool and Slows Down Replenishment. *Int. J. Mol. Sci.* **2022**, *23*, 6429

**DOI:** 10.3390/ijms24021561

**Published:** 2023-01-13

**Authors:** Chris S. Mesnard, Cody L. Barta, Asia L. Sladek, David Zenisek, Wallace B. Thoreson

**Affiliations:** 1 Department of Ophthalmology and Visual Sciences, Truhlsen Eye Institute, University of Nebraska Medical Center, Omaha, NE 68198, USA; 2 Pharmacology and Experimental Neuroscience, University of Nebraska Medical Center, Omaha, NE 68198, USA; 3 Department of Cellular and Molecular Physiology, Yale University, New Haven, CT 06510, USA

The authors wish to make the following corrections to this paper [1]: In the original publication, there was a mistake in Figure 6 as published. We reversed the data in panels C and D, plotting B-wave latency data as A-wave amplitude in panel C and A-wave amplitude as B-wave latency in panel D. The corrected Figure 6 appears below. 

The authors apologize for any inconvenience caused and state that the scientific conclusions are unaffected. This correction was approved by the Academic Editor. The original article has been updated.

## Figures and Tables

**Figure 6:**
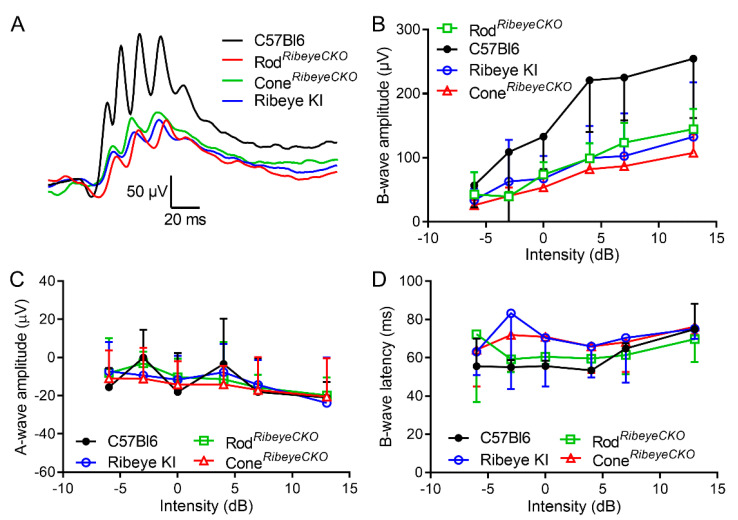
Photopic ERG b-waves were reduced equally in the cone*^Ribeye^*^CKO^, rod*^Ribeye^*^CKO^, and *Ribeye* KI retinas. (**A**) Example waveforms. (**B**) B-wave amplitude as a function of flash intensity measured in the C57Bl6J (*n* = 5 mice), cone*^Ribeye^*^CKO^ (*n* = 5), rod*^Ribeye^*^CKO^ (*n* = 5), and *Ribeye* KI retinas (*n* = 4). (**C**) A-wave amplitude as a function of intensity. (**D**) B-wave latency vs. intensity.
